# Engineering Biology to Construct Microbial Chassis for the Production of Difficult-to-Express Proteins

**DOI:** 10.3390/ijms21030990

**Published:** 2020-02-02

**Authors:** Kangsan Kim, Donghui Choe, Dae-Hee Lee, Byung-Kwan Cho

**Affiliations:** 1 Department of Biological Sciences, Korea Advanced Institute of Science and Technology, Daejeon 34141, Korea; kskim2474@kaist.ac.kr (K.K.); robinald@kaist.ac.kr (D.C.); 2 KAIST Institute for the BioCentury, Korea Advanced Institute of Science and Technology, Daejeon 34141, Korea; 3 Synthetic Biology & Bioengineering Research Center, Korea Research Institute of Bioscience and Biotechnology, Daejeon 34141, Korea; dhlee@kribb.re.kr; 4 Intelligent Synthetic Biology Center, Daejeon 34141, Korea

**Keywords:** heterologous protein expression, difficult-to-express proteins, synthetic biology, systems biology, genome reduction, genome synthesis.

## Abstract

A large proportion of the recombinant proteins manufactured today rely on microbe-based expression systems owing to their relatively simple and cost-effective production schemes. However, several issues in microbial protein expression, including formation of insoluble aggregates, low protein yield, and cell death are still highly recursive and tricky to optimize. These obstacles are usually rooted in the metabolic capacity of the expression host, limitation of cellular translational machineries, or genetic instability. To this end, several microbial strains having precisely designed genomes have been suggested as a way around the recurrent problems in recombinant protein expression. Already, a growing number of prokaryotic chassis strains have been genome-streamlined to attain superior cellular fitness, recombinant protein yield, and stability of the exogenous expression pathways. In this review, we outline challenges associated with heterologous protein expression, some examples of microbial chassis engineered for the production of recombinant proteins, and emerging tools to optimize the expression of heterologous proteins. In particular, we discuss the synthetic biology approaches to design and build and test genome-reduced microbial chassis that carry desirable characteristics for heterologous protein expression.

## 1. Introduction

Heterologous protein expression by means of the genetically engineered prokaryotic host has made available a wide spectrum of recombinant proteins that are otherwise confined to limited natural origin, in a scalable and cost-effective manner. Marked with the expression of an active human protein somatostatin in *Escherichia coli* back in 1977 [1] followed by monumental success in the expression of human insulin [2] and its marketization [3], much focus has been shed on microbe-based protein expression system for its versatile nature and potential for large-scale production. Demands for commercial proteins have also become increasingly diverse—characterized by extensive variations in biochemical and structural properties. These include, but are not limited to, therapeutics for clinical treatment [4,5], antibodies for diagnostics [6], and enzymes for industrial use [7]. Such divergence in biochemical, structural, and functional aspects of recombinant proteins means that there are a vast number of factors to consider before achieving functional expression of the recombinant proteins in bacteria.

While bacterial systems are capable of expressing a wide spectrum of heterologous proteins in their functional forms [4], there remain imminent challenges and limitations in utilizing this system to its fullest. Every so often, heterologous expression of proteins alien to the host system poses significant difficulties and requires extensive optimization steps to tame. For instance, problems highly recursive in heterologous protein expression include improper folding of target proteins, especially those of higher eukaryotes that render the protein to lose its native function. This is largely attributed to host factors such as differences in cytoplasmic redox potential that interfere with disulfide bond formation [8], differences in codon usage, and repetitive DNA sequences that affect protein translation and subsequent protein folding [9]. Furthermore, the functional expression is largely implicated with the size and characteristics of the heterologous protein. It was reported that proteins with large molecular weight or those that harbor several membrane domains pose a far greater tendency to form insoluble aggregates and are prone to proteolysis [10]. Moreover, additional challenges include mimicking eukaryotic post-translational modifications (such as glycosylation) [11], production of harmful endotoxins (*E. coli*) [12], reduced cell viability resulting from unwanted by-products [13], and low protein yields [14]. To tackle these obstacles, different recombinant DNA technologies, including manipulation of gene expression control [15,16,17], directing proper bond formation and protein folding [18,19], interfering host metabolic pathways [20,21], random or directed evolutions of bacterial strains or enzymes [22], and series of other methods have been systematically employed (summarized in [23]). While these engineering strategies already provide practical solutions for the expression of various protein forms, the engineered expression systems can further be optimized to achieve a better performance. 

Functional protein expression has met a dramatic shift paralleled with the advances in systems and synthetic biology; that is, data-driven understanding in systems biology has provisioned a knowledge-based framework on which to rationally design, build, and test biological entities in an increasingly sophisticated manner [24]. For instance, the following have altogether enabled characterization of the bacterial host and recombinant proteins at the systems level: a large influx of high-throughput information on DNA, RNA, proteins, and metabolites that enabled visualization of the global landscape of transcriptional and translational processes [25,26]; availability of robust computational tools and in silico models streamlining detailed analysis and prediction of the cellular networks; and rapid advances in DNA synthesis, assembly techniques that facilitated the process to build and test the designed biological systems [27,28]. Such new schemes provide one possible way around biological uncertainties; that is, by systematically eliminating genetic redundancies, it is expected to minimize the confounding effects of unknown genetic elements and to reduce metabolic interference from native metabolic pathways while endorsing more predictable regulation of cellular functions [24]. This, in essence, would help to further improve the performance of the extant microbial cell factories. In addition, the design and synthesis of minimal genomes can be paralleled with re-engineering efforts to repurpose a cell to execute certain functions, including genome recoding to expedite production of unnatural polypeptides [29], and refactoring genes to make transcriptional and translational regulation more controllable [30]. Hosts of different microbial synthetic minimal genomes have been published to date (reviewed in [31]), with many harboring genetic and phenotypic traits to better house and support biosynthesis of heterologous proteins and commodity biomolecules.

## 2. Heterologous Expression of Biologically Functional Proteins by Bacteria 

**Figure 1:**
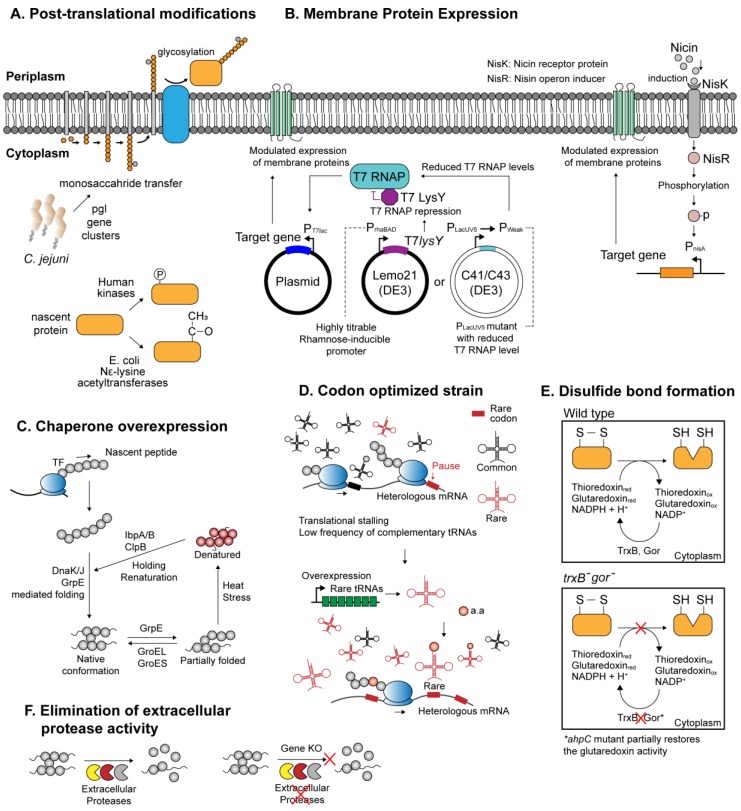
Notable characteristics of microbial chassis. (**A**) Post-translational modification systems involving glycosylation, phosphorylation, and acetylation; (**B**) various protein expression systems catered for the expression of membrane proteins; (**C**) simplified mechanism of the peptide folding mediated by the molecular chaperones; (**D**) expression system optimized for the expression of the heterologous proteins with high rare codon frequencies; (**E**) genetically engineered *E. coli* strain (*trxB^-^*, *gor^-^*) enabling the formation of disulfide bridges within the cytoplasm; (**F**) increasing recombinant protein yield by genetically deleting extracellular protease genes.

Production of recombinant proteins into a biologically functional form necessitates robust expression systems that can reliably execute proper folding, precise post-translational modifications, and efficient translocations while maintaining cellular viability (Figure 1). In recent years, prokaryotic expression systems have been an effective means to deliver functional proteins at high yields. Genomic, genetic, and biochemical aspects of the microbial systems have been extensively explored, culminating in series of genetically engineered microbial chassis with features desirable for heterologous protein expression. Notwithstanding these efforts, no single expression system can support all existing recombinant proteins, and this is especially the case for difficult-to-express proteins, which often require highly specialized strain engineering. 

### 2.1. Engineered E. coli for Wide Array of Recombinant Proteins

Over 50% of the recombinant proteins registered to date (Protein Data Bank [32]) are of eukaryotic origin, with more than 90% of them being produced in prokaryotic expression systems [32,33]. Among all recombinant expression hosts at work, *E. coli* is by far the most favored strain, owing to its outstanding genetic tractability, relative ease of cultivation, and innate capacity to accommodate and express exogenous proteins. Specifically, it is the model microbe with the most extensively characterized genome, transcriptome and translatome architectures, and underlying regulations [25,34]. In addition, the availability of a vast repertoire of synthetic biology tools such as libraries of promoters, ribosome-binding sites (RBS) and 5′-untranslated regions (5′-UTRs), expression vectors, and synthetic circuits effectively streamline its genetic manipulation [35,36,37,38,39]. In aspects of protein productivity, *E. coli* was reported to dedicate nearly 40% of its dry cell weight entirely for recombinant protein in fed-batch culture conditions [40], and are able to functionally express a wide spectrum of non-glycosylated proteins. Such inherent features of *E. coli* have much been exploited in the industry for mass-production of many commodity proteins [41].

Even so, securing high-purity, high-yield recombinant proteins, especially those of eukaryotic origin, has remained challenging in *E. coli* due to constraints inherent to cellular physiology and translational regulations. For instance, just as many prokaryotic expression systems, *E. coli* lacks post-translational modification machineries required for functional expression of proteins in eukaryotic origins, such as glycosylation [11]. This could present a serious drawback in prokaryote-based expression systems, considering that more than 50% of eukaryotic proteins are predicted to be glycosylated [42]. Other classes of post-translational modifications highly prevalent across eukaryotic proteins include phosphorylation and acetylation [43]. These modifications contribute as much, if not more, to proper protein folding [44], and endows proteins with important functionalities (Table 1). 

For instance, the finding that a gram-negative bacterium *Campylobacter jejuni* harbors a functional *N*-linked glycosylation pathway encoded by *pgl* gene cluster, which can be reconstituted and expressed in *E. coli*,has widened the scope of prokaryotic-based heterologous protein expression to various glycoproteins (Figure 1A and Table 1) [45,62]. Subsequently, several technical limitations arising from the differences in bacterial and eukaryotic *N*-glycosylation systems, such as different glycan specificity [63], were circumvented with novel engineering attempts, hence further refining the applicability of the glycosylation-competent *E. coli* [63,64]. On the other hand, phosphorylation-competent prokaryotic expression system has been developed by introducing mammalian kinases into the host cell. Several human kinases coexpressed with recombinant protein in *E. coli* were shown to successfully phosphorylate the target proteins in their native patterns (Figure 1A and Table 1) [46,65]. Furthermore, a number of expression systems have been devised in *E. coli* to deliver proteins with intact acetylation patterns. For example, *E. coli* is known to harbor multiple endogenous *N*^ε^-lysine acetyltransferases (KATs), which catalyzes acetylation of lysine side chains embedded within proteins [66]. It was demonstrated that the native acetylation machineries in *E. coli* can be exploited to effectively acetylate human thymosin α1, suggesting that production of acetylated proteins is likely achievable in recombinant microbes (Figure 1A and Table 1) [47]. Similar to the method used in protein phosphorylation, coexpression of eukaryotic acetylation enzymes, such as NatA or NatB complex from yeast, was shown to provide an effective means for amino-terminal acetylation (Table 1) [48]. Other, more complicated approaches involved site-directed incorporation of acetyllysine, an acetyl-derivative of lysine, on target recombinant protein using a three-plasmid expression system (Table 1) [49]. Each of the three plasmids expresses (i) a target gene with lysine codons replaced with amber codons; (ii) a suppressor tRNA that recognizes the amber codon; (iii) an aminoacyl-tRNA synthetase evolutionarily engineered to charge *N*^ε^-acetyllysine to the suppressor tRNA. By suppressing the action of native deacetylase, the recombinant *E. coli* managed to synthesize manganese superoxide dismutase with site-specific acetylation.

Inside a cell, newly synthesized peptides are highly susceptible to be misfolded, either as the result of aberrant translational termination or due to abrupt changes in protein conformation in response to environmental stresses. The molecular chaperones play a crucial role in maintaining cellular proteomic integrity by guiding proper folding of nascent proteins into their native conformations and directing refolding of misfolded or aggregated proteins in the cytoplasmic milieu [19]. The chaperone systems most relevant for de novo protein folding include trigger factor (TF), GroEL system, DnaK system, GrpE, and ClpB, each with a distinct but partially overlapping mode of actions. For instance, de novo folding of polypeptides emerging from the ribosome is aided by TF, DnaK system, and subsequently with GroESL system to reach a native conformation (Figure 1C) [19]. In a synthetic biology perspective, this ubiquitous protein folding machinery is an important molecular asset to tackle the problem in heterologous expression of recombinant proteins—protein misfolding. In *E. coli* and other prokaryotic systems, accumulation of overexpressed recombinant proteins in the form of misfolded, insoluble aggregates is rather common [8]. The high incidence of misfolding among the overexpressed recombinant proteins may reflect the inherent upper limit of the host chaperone capacity. Efforts have been made to overexpress single and multiple combinations of chaperones native to the host, which often resulted in meaningful improvements in the net solubility of recombinant proteins [67,68]. Likewise, the co-overexpression of heat-shock chaperones and ClpB chaperone that serve to refold the insoluble aggregates also enhanced the solubility of numbers of recombinant proteins (Table 1) [50,68]. In addition, it was shown that the protein solubility can further be augmented by allowing a prolonged incubation time for in vivo protein–chaperone interaction, which increased the solubility of an array of heterologous proteins for up to 42-folds [50].

On the other hand, reducing conditions in the prokaryotic cytoplasm may inflict biochemical perturbations on nascent polypeptides, interfering with proper disulfide bond formation and rendering the afflicted proteins insoluble [8,69]. Disulfide linkage represents an abundant group of a structural scaffold in protein (with more than one-third of eukaryotic proteins predicted to harbor disulfide bond [70]) and is implicated in diverse aspects of protein functionalities. However, disulfide bond formation in the prokaryotic cytoplasm, such as that of *E. coli*, is disturbed by the presence of numerous reductases and other reductants [69]. Instead, native proteins that necessitate disulfide bonds are subjected to periplasmic secretion with N-terminal signal peptides directing their translocation. The oxidizing periplasmic space provides an amenable medium for oxidation of cysteine residues, and disulfide bond formation is further expedited by the presence of thiol–disulfide oxidoreductases enzymes, such as DsbABCD [70]. Hence, one approach to express disulfide-positive recombinant proteins in the microbial periplasm involves fusion with bacterial signal peptides, which is covered in detail elsewhere [71]. An alternative approach is to genetically engineer a bacterial host to reconfigure the cytoplasmic environment conducive for disulfide bond formation. For example, two pathways—glutaredoxin and thioredoxin pathways—are responsible for maintaining the reducing cytoplasm in *E. coli* (Figure 1E) [72]. Several collective findings have led to an *E. coli* expression system *E. coli* FA113, catered for disulfide bond formation (Table 1) [51,72,73] (widely known as Origami™). More recently, CyDisCo (cytoplasmic disulfide bond formation in *E. coli*) system revealed that disulfide bond formation can be reliably achieved in an otherwise wild-type *E. coli* cytoplasm (Table 1) [52]. CyDisCo strain harbors genes that encode mitochondrial thiol oxidase Ervp1 of yeast origin, and the human protein disulfide isomerase (PDI). In addition, this system can be brought up to a large-scale, fed-batch culture yielding considerable titer for human antibody, interleukins, and growth hormones [74]. 

Membrane proteins present yet another challenge for heterologous expression. Arising in complex multiple membrane-spanning domains, membrane proteins exhibit extensive variations in terms of structural, chemical, and functional representations. Problems recurrent in heterologous expression include (i) inclusion body formation and cell death resulting from saturation of membrane protein synthesis pathway and (ii) low-level expression [53]. Several expression systems more tolerant to membrane protein expression have been developed. For instance, *E. coli* C41(DE3) and C43(DE3) isolated from BL21(DE3) mutants are able to express membrane proteins more efficiently than the wild-type BL21(DE3) (Figure 1B and Table 1) [53]. Subsequent assessment of individual mutations in C41(DE3) revealed that increased tolerance toward the toxic expression is ascribed to the mutations in the P*_lac_*_UV5_ promoter which reduced T7 RNA polymerase levels upon isopropyl β-D-1-thiogalactopyranoside (IPTG) induction in C41(DE3) [75]. This is consistent with the fact that the overexpression of heterologous membranous proteins is especially toxic in the prokaryotic hosts and often suffers low protein yield. In this sense, the reduced expression strength of the P*_lac_*_UV5_ promoter in C41(DE3) strain may have helped reach a compromise between protein productivity and cellular fitness. The case of C41(DE3) sheds light on the important rule of thumb that tight control of gene expression is imperative to obtain membrane proteins while preventing undesirable outcomes such as cell death. In this respect, *E. coli* Lemo21(DE3) is a preferred host of choice for membrane protein expression which allows fine-tuning of overexpression intensity (Figure 1B and Table 1) [54]. Specifically, Lemo21(DE3) is isogenic to BL21(DE3), except that it harbors an expression vector that contains T7 lysozyme (T7 RNA polymerase inhibitor) tightly regulated by a rhamnose promoter which confers a highly titratable, broad range of expression control [76]. 

### 2.2. Bacillus Subtilis as A Versatile Host with Highly Efficient Protein Secretion Systems

Being an efficient producer of many desirable enzymes, *Bacillus* species have long been used as an efficient workhorse for the production of various commodity molecules, including industrial enzymes [77] and pharmaceuticals [78]. Excellent genetic tractability, robust growth in laboratory settings, and the vast availability of genetic tools and expression vectors comparable to those of *E. coli* made them highly pliable for genetic modifications and culture [79]. Notably, *Bacillus* species are known for their excellent protein secretion systems. As gram-positive bacteria with a single sheath of membrane separating between cell and outer environment, proteins can be readily secreted into the culture medium in biologically active forms via specialized secretion machineries. This confers a great advantage compared with *E. coli* expression system, where the overexpressed proteins tend to accumulate inside the cellular compartments, often in the form of insoluble aggregates. This defining trait in *Bacillus* considerably reduces the time and cost associated with downstream purification or extraction processes. As a side note, lack of apparent pathogenicity (viewed as Generally Recognized As Safe organism) gives an extra perk in its utilization, obviating the cumbersome and costly processes to remove toxic impurities such as lipopolysaccharide (LPS) or endotoxin in *E. coli* [80]. Importantly, several *Bacillus* strains, including *B. subtilis,* harbor large-scale fermentation capacity and are used for bulk manufacturing of industrially used proteins. These characteristics, along with several others, make *Bacillus* a robust production platform for heterologous proteins. 

As it is said, the choice of *Bacillus* is almost exclusively ascribed to their highly efficient secretion system, which, given the right condition, yields as high as tens of grams per liter of proteins [81]. It is now understood that most of the protein exports are mediated via the general secretory (Sec) pathway, with the remaining few transported by a set of more specialized transport systems including twin-arginine translocation (TAT) pathway [82]. These existing modalities of protein export can be genetically engineered to facilitate secretion of heterologous proteins. For instance, the absence of compatible secretory proteins required for secretion of heterologous proteins in *B. subtilis* can be complemented by coexpressing a secretory protein SecB, along with genetic modifications in the host translocase component SecA to effectively bind SecB [83]. In another instance, engineering the SecA-dependent secretion pathway alone can double the productivity of recombinant proteins [84]. However, one drawback of such signal-peptide-mediated protein export is that the process requires complex interplay of multiple cellular components, which requires an extensive optimization for a large-scale protein secretion. Alternatively, several reports have shed light on nonclassical protein secretion systems that mediate translocation of proteins that lack signal peptides as a more straightforward means to direct protein secretion [85]. Specifically, all routes by which the cytoplasmic proteins without apparent secretion motifs travel out into the extracellular space are termed as *nonclassical secretion pathway* due to their largely elusive mechanism of transport [86]. Notably, in analogy to fusion tags, physically linking such nonclassically secreted proteins to heterologous expression target was shown to functionally mimic the role of signal peptides, enhancing the secretion rates of fusion proteins without compromising their biological functions [85]. This suggests that nonclassically secreted proteins may serve as a novel molecular tool for effective secretion of target proteins.

At the same time, *Bacillus* strains are also known for their profuse secretion of intracellular and extracellular proteases, with at least seven different extracellular proteases reported to date in *B. subtilis* [87]. While secretory proteins native to *Bacillus* exhibit resistance against the proteolytic activity of the proteases, most recombinant proteins are highly prone to proteolysis and are often rendered nonfunctional [88]. Protease-deficient *Bacillus* strains, such as *B. subtilis* derivative WB600, have thence been genetically engineered to circumvent host-mediated proteolysis (Figure 1F and Table 1) [56], though it only remained a partial success due to the persisting proteolytic activity [57]. Nonetheless, WB600 yielded higher productivity of recombinant proteins including β-lactamase [56], streptokinases [89], and several others. Subsequent removal of the remaining protease-encoding genes *vpr* and *wprA* in WB600 has led to an increased stability of the expressed protein molecules [90]. Several proteins still suffered from proteolytic degradation despite near-complete absence of the proteolytic activity in the protease-deficient *B. subtilis* strains. It was suggested that proteins in their prefolded state are highly susceptible to proteolytic activity, and finding a way to expedite folding of the nascent proteins would significantly improve their half-life post-secretion. One way to facilitate protein folding is by increasing the availability of metal cations such as Mg^2+^, Ca^2+^,^,^ and Fe^3+^ around the cell membrane microenvironment. These ions are also found in several protein species, including recombinant protective antigen (rPA), and serve as essential components to promote protein folding (Table 1) [57]. 

The development of novel genetic engineering tools and techniques are further refining the capacity and applicability of the existing *Bacillus* heterologous expression systems in recombinant protein production [91]. Several studies provide guidelines to utilize the existing techniques to their best efficiency and compatibility. One such report describes a combinatorial optimization scheme involving fine-tuning of promoter strength, translation, and folding efficiency in a protease-deficient *B. subtilis*, which altogether led to a ninefold increase in the production of human fibroblast growth factor 21, whose soluble expression is difficult due to protein aggregation [14]. Other recent technical aspects of recombinant protein expression in *Bacillus* have been reviewed elsewhere [92].

### 2.3. Lactococcus Lactis for the Expression of Recombinant Membrane Proteins

Having been widely used in dietary industries as a fermenting bacterium, *Lactococcus lactis* is a gram-positive, lactic-acid-producing bacterium with an emerging role in heterologous protein expression. It earned its recognition following the functional secretion of the heterologously expressed cytokine interleukin-10, which alleviated the symptoms of the inflammatory bowel disease (IBD) in the IBD model mouse in vivo [93]. Several characteristics of *L. lactis* expression system were deemed desirable for development and production of recombinant proteins and therapeutics; these include (i) single-membraned prokaryote with efficient protein secretion system; (ii) negligible extracellular proteolysis activity; (iii) generally recognized as safe (GRAS) strain free of endotoxins; (iv) scalable fermentation capacity; and (v) the availability of both inducible and constitutive genetic control systems such as the nisin-inducible expression (NICE) system. 

The NICE system is a popular expression system of choice among the *Lactococcal* strains for the expression of a range of proteins, including hard-to-obtain membrane proteins (Figure 1B and Table 1). This system constitutes a two-component signal transduction system of *nisK* and *nisR*, which together activate the promoters in *cis* in response to subinhibitory concentrations of nisin [58]. In particular, an *L. lactis* NZ9000 strain that incorporates *nisK* and *nisR* has been developed [58] and along with its derivatives, is in active use for recombinant protein expression [94]. This system, along with others, has been used to produce various prokaryotic as well as eukaryotic proteins of animal and plant origin [95,96,97], with disulfide bonds intact [98]. Of particular note, *L. lactis* expression system has been proven to be highly efficient in membrane protein expression, where the expressed membrane proteins are localized to the cytoplasmic membrane and the use of mild detergents can readily solubilize these membrane proteins [94]. It is also worth noting that the quality of protein conformation and solubility of recombinant proteins in *L. lactis* are closely coupled to culture conditions including temperature and production time [99]. While synthetic biology tools available for *Lactococcus* are not as diverse as those of *E. coli* or *Bacillus*, the engineered *L. lactis* strain and its derivatives nonetheless exhibited an excellent potential for the production of difficult-to-express proteins. With growing availability of genetic tools and techniques [100], it is expected to serve as a robust microbial chassis for recombinant protein expression. 

### 2.4. Extremophiles as Alternative Protein Expression Systems

Extremophiles refers to microbial cells that function optimally in environments that are extreme for common life forms, as characterized by harsh temperature, salinity, and pH. Some of the adaptive features harbored by these extremophiles hold potentially desirable biotechnological values. For instance, the cytoplasmic compartments of moderate halophiles are densely packed with organic compounds or osmolytes as part of an adaptive response to cope with high-salt conditions [101]. This led to speculation that the moderate halophilic cytoplasm could confer higher protein folding efficiency compared to other mesophiles like *E. coli* [102,103]. It was empirically demonstrated that several halophiles successfully accommodate mammalian proteins in a soluble state. These include functional expression of human ß_2_-adrenoceptor and mammalian olfactory receptors in *Haloferax volcanii* [59,60] (See Table 1) and human brain serine racemase from *Halomonas* sp. and *Chromohalobacter salexigens* [104]. Furthermore, the expression of proteins with a high content of acidic amino acids in halophilic bacteria are under vigorous investigation as potential protein fusion partners to expedite solubilization of recombinant proteins [103].

Thermophiles are increasingly viewed as an alternative prokaryotic expression system that would complement the inherent shortcomings of mesophilic bacteria. Thermophilic microbes represent species of bacteria and archaea that thrive at high temperatures, usually more than 45 °C [105]. The biotechnological importance of thermophiles is appraised in their capacity for high-temperature bioprocesses and functional expression of thermostable enzymes including DNA polymerases and many other biocatalysts with industrial significance [106]. As for the thermostable enzyme production, although several thermostable proteins have been successfully expressed in *E. coli* hosts [107,108], they are largely limited in their extent to simple monomeric proteins that come without strict requirements for cofactors, additional modifications, or processing [106]. In fact, a significant portion of thermophilic proteins, especially those of hyperthermophilic origin, require high temperature and the presence of specific chaperones or other cofactors for proper protein folding [109]. This signifies that the expression of thermostable enzymes, especially heteromultimeric enzymes, would require a cell factory closely related to native thermophiles.

Despite its significance, heterologous expression of thermostable proteins in thermophilic hosts is still an underexplored field of expertise. One limitation is that genetically tractable thermophiles are relatively scarce compared to other mesophilic bacteria. This is largely ascribed to harsh laboratory culture conditions—high temperature, pH, and salinity—that render most of the temperature-sensitive molecular markers obsolete [105]. Regardless, significant efforts have been put forward to develop genetic systems orthogonal to thermophilic bacteria and archaea in the past decade (summarized in [105,110]), enabling overexpression of homologous and heterologous proteins in the thermophiles. For instance, heat- and arabinose-inducible promoters in the hyperthermophilic archaeon *Sulfobolus solfataricus* have been used for the soluble expression of proteins by up to 1 mg per liter [109]. In another instance, discovery of a novel inducible promoter system in a thermophilic bacterium *Geobacillus kaustophilus* HTA426 led to functional expression of recombinant enzymes, which were otherwise insoluble in mesophilic hosts, for up to 59 mg per liter [61] (Table 1). However, the current status of thermophilic expression hosts is far from being ideal compared to that of highly optimized mesophiles which yield as much as tens of grams per liter of recombinant proteins [81]. Yet, with steady improvements in the molecular genetics of thermophiles, they are highly anticipated as emerging hosts for the overexpression of much-coveted thermostable proteins.

## 3. Heterologous Protein Expression by Systematically Engineered Bacteria

### 3.1. Concept and Overview of Synthetic Minimal Genome

Some of the defining characteristics of heterologous expression host can be described in two distinct, but not mutually exclusive, perspectives. First, contemporary ideas on expression hosts mainly describe them as versatile biological systems capable of accommodating and supporting the functional expression of exogenous genetic elements in a controllable manner [111]. Following the rise of multi-omics technology, an emerging view on an ideal expression host is characterized by microbial strains that harbor simplified genetic and metabolic networks amenable to prediction and control, through which an efficient biosynthesis of desired products can be achieved [112]. The former and the latter statements represent the popular ideas in the field of synthetic and systems biology in regard to microbial chassis strains tailored for heterologous expressions. The shared perspectives of synthetic and systems biology together brought into realization the concept of minimal genomes along with their proposed advantages across diverse fields of applications, including expression of heterologous proteins. A growing number of reports already highlight the advantages of genome-reduced microbial strains outperforming their wild-type counterparts in terms of productivity of target proteins [113] and desired biomolecules [114], which result from the elimination of unfavorable features for protein expression and rewired metabolic networks redirected to produce heterologous proteins. In this section, we attempt to provide a brief overview of the concept, design, and mechanism underlying synthesis of the minimal genome. 

**Figure 2:**
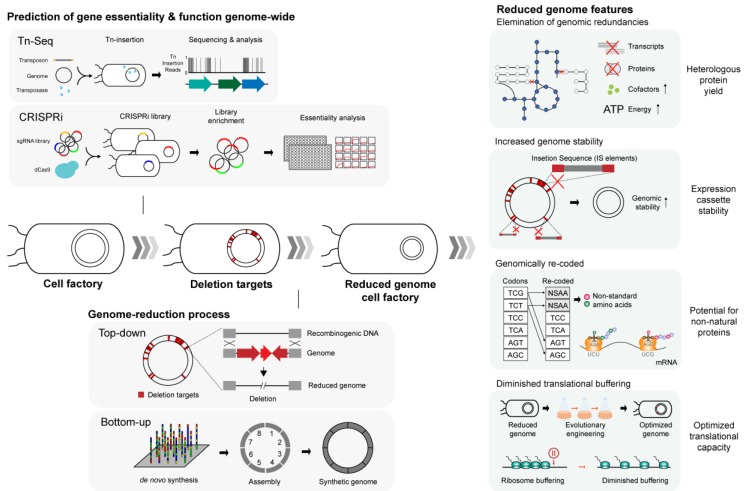
Illustration of the process behind genome streamlining and several notable traits of the genome-reduced strains with relevance to heterologous protein expression.

Availability of gene-essentiality information facilitates the genome reduction process in that it provides a shortlist of genetic elements that could be targeted for removal, circumventing the laborious trial-and-error gene deletion approaches. With gene essentiality information in hand, genome reduction can proceed in either a bottom-up or top-down manner (Figure 2). The bottom-up approach entails designing and building an artificially synthesized genome from scratch. Laboratory-made genetic fragments that constitute a section of the target genome are assembled into larger segments by means of enzymatic assembly [27] and yeast homologous recombination into the partial or whole genome [115]. This way, the minimal genome of *Mycoplasma genitalium* and *Mycoplasma mycoides* were de novo assembled into biologically functional forms [115,116]. More recently, the genome-reduced *E. coli* MDS42 was de novo assembled and synthesized into a genome-recoded version [117] using newly developed homologous recombination methods that facilitate the iterative replacement of large stretches of DNA strands in a highly efficient manner [118]. Implications of the synthetic genome in heterologous protein expression are centered on genome-scale codon replacements, where incorporation of synthetic or rare codons may help facilitate synthesis of unnatural polypeptides [119] and heterologous proteins of eukaryotic origin that grossly differ in their codon content. Details on heterologous protein synthesis using synthetic genome will be discussed in subsequent sections. 

Conversely, top-down genome-reduction proceeds with systematic removal of nonessential genetic elements starting from an intact genome of the target organism. Typically, homologous recombination mediated by λ red [120] or site-specific Cre-*loxP* [121] and Flp-FLT [122] recombination methods are employed in the process of genomic deletions. Due to relative ease of underlying procedures and cheaper costs, a larger number of top-down genome reduction projects have been published to date as compared with that of bottom-up synthesis (Table 2). Strains subjected to top-down genome-reductions to date include *E. coli* [123,124,125,126,127], *Streptomyces* strains [114,128,129,130,131,132,133], *B. subtilis* [92,134,135,136,137,138], *L. lactis* [139], *Pseudomonas putida* [140,141], *Corynebacterium glutamicum* [142], and a yeast strain [143]. This strategy enabled the removal of unnecessary proportions of genome at large scale without causing palpable defects in the target organisms (except in a few instances [135,144]) and sometimes yielded improved cellular performances in terms of growth rates, cell density, transformation efficiency, and protein productivity compared with their wild-type counterparts. With an unintended discovery of the desirable properties that synthetic minimal genomes may convey in microbial production, several studies have explored their potential for industrial applications for recombinant protein production [145,146,147,148]. The following sections have been outlined to elaborate in detail on cellular mechanisms underlying improved performance in genome-reduced strains and some of the practical examples of the minimal genome expression system, and also to discuss potential applications of genome-reduced strains in heterologous protein expressions. 

### 3.2. Applications of the Minimal Genome: from Gene Essentiality to Protein Production 

#### 3.2.1. Construction of Genome-Reduced Microbial Strains 

The essence of genome reduction lies in identifying genes that are indispensable for sustaining cell viability. Previous attempts to determine gene essentiality involved a priori predictions derived from in silico comparative genomics in search of homologs and paralogs among remotely related species [150] or systematic inactivation of individual genes from the bacterial genome [151,152]. However, these line of efforts to define essential genes only remained a partial success, as it turned out that evolutionary gene conservation is a poor indicator of gene essentiality [150], and that the nature of gene essentially is highly condition-specific, rendering it almost infeasible to determine using the earlier experimental designs that are extremely time-consuming and laborious [151,152]. Today, the construction of minimal genome is usually accompanied by high-throughput techniques such as transposon mutagenesis sequencing (Tn-Seq) or CRISPR-dCas9, which present a reliable systems-level reference on genome-wide gene essentiality information (Figure 2) [153,154], allowing rational selection of genomic regions for targeted removal. Genome reduction then serves as a testbed to further evaluate the essentiality of the targeted genes or genomic regions and classify them into subcategories in accordance with resulting cellular viability and changes in growth rates ensuing from the targeted deletion (Figure 2) [116]. 

Apart from its use in assessing genome-scale essentiality, potential uses of minimal genome strains extend to metabolic engineering and protein production. Although there exists a wealth of information on many cellular processes at different subsystems, it was showcased to be extremely challenging to understand the regulatory networks integrated across the subsystems. Synthetic lethality presents a prominent example of unknown regulatory effects, where two independent genes or genomic regions that are otherwise nonlethal when deleted individually are lethal upon simultaneous deletion [126]. Similarly, the deletion of gene candidates that are thought to be redundant or remotely related to certain physiological responses can result in unintended consequences in bioproduction [155]. Hence, the scheme behind minimal-genome-driven metabolic engineering revolves around the idea that simplification of the genome would lead to bacterial chassis with fewer unknown regulations and lesser interference on heterologous pathways, thus allowing more predictable modeling of the chassis of interest [156].

#### 3.2.2. Effect of Genomic Stability on Protein Production 

Several genome-reduced strains, by both chance and intent, showcased some of the phenotypes highly favored for the production of heterologous proteins. First, high-fidelity expression of heterologous proteins is ensured by the stability of the genome or expression plasmids that harbor target genes or pathways. Many microbes of industrial use, including *E. coli*, are often plagued by the activation of mobile DNA elements that could randomly inactivate the heterologous expression system [157]. Typically, the expression of heterologous proteins demands a significant portion of metabolic resources from the recombinant host, diverting cellular resources such as ATP, precursors for biosynthesis, and translational machineries away from the host’s innate metabolism, hence imposing a metabolic burden on the host microbe [28,158]. In this context, insertion sequence (IS)-mediated mutagenesis serves as an adaptive response to alleviate the metabolic burden by disrupting the expression of foreign proteins, which, in turn, adversely affects the target protein production [157]. The genomes of several *E. coli* genome-reduced strains were shown to be far more stable compared with their deletion-free parental strains (Figure 2) [124,127,149]. This is largely owing to the complete or partial excision of mobile DNA elements, in particular, insertion sequences (ISes) from the genome, ameliorating the possibility of IS-mediated mutagenesis. For instance, it was empirically demonstrated that the first IS-free *E. coli* strain MDS42 with 14% genome reduction restored the yield of a chimeric fusion protein and stably propagated adeno-associated viral vector plasmids that are otherwise frequently inactivated and destabilized in the IS-positive parental MG1655 strain (Table 2) [124]. Similarly, another IS-free genome-reduced *E. coli* strain MS56 exhibited similar genomic and plasmid stability while exhibiting cellular fitness comparable to that of the parental strain, despite a staggering 23% reduction in the genomic content (Table 2) [127]. Succeeding studies have focused on practical applications of the IS-free, genome-reduced strain for the production of commodity biomolecules. *E. coli* MDS42 was re-engineered with additional genetic manipulations to stably overexpress toxic recombinant protein methyltransferase in its functional form [146] and boosted production of essential amino acid, L-threonine, by 83% [147]. 

#### 3.2.3. Increased Availability of Cellular Resources 

Along with the genetic fidelity of the heterologous expression system imparted by the removal of mutation-causing cellular machineries, many other factors may account for the improved recombinant protein yield in the genome-reduced expression system. In particular, enhanced biosynthesis of desired protein is largely attributable to the removal of innate but redundant metabolic pathways, which prompt to redirect energy, precursor pools, cofactors, and transcriptional and translational machineries toward heterologous metabolic pathways [24,28,128,158]. The metabolic burden imposed by expressing heterologous genes arises from competition for shared but limited cellular resources between exogenous genes and host metabolism. Thus, removal of host genes that are nonessential translates to an overall decrease in the resource cost spent on now-absent host metabolism, which then reduces the net metabolic load [159] and allows for efficient resource utilization and protein translation (Figure 2) [24,137,145]. In addition, reduced genomic complexity in the minimal cells may help to curtail regulatory interference with the targeted or exogenous metabolic pathways [114], reducing the number of compounding variables that pose unknown effects on heterologous expression. Such shared metabolic predispositions of the genome-reduced strains offer a fascinating platform for the bioproduction of numerous valuable commodity molecules, including heterologous proteins. 

Empirically, *B. subtilis* MBG874 strain that underwent a 20% genome reduction exhibited increased productivity of episomally expressed proteins up to 2.5-fold higher than that of the wild-type *B. subtilis* 168 (Table 2) [113]. In another instance, mini-*Bacillus* strains that retained only two-thirds of the *B. subtilis* 168 genome, with extra re-engineering effort, were able to stably express four individual staphylococcal antigens that are recalcitrant for heterologous protein expression in the existing *B. subtilis* chassis (Table 2) [145]. Genome-streamlined *L. lactis* chassis NZ9000 specializing in the production of many membranous and disulfide-containing proteins also exhibited superior growth rate, biomass yield, and nutrition utilization capacity [139]. Importantly, 2.8% of genome reduction targeting the prophages and their cognate genes in *L. lactis* significantly improved the production of the model recombinant proteins, leucocin C and the red fluorescence protein (RFP). Phenotype screenings of the target proteins showed up to 2.5-fold increase in the inhibiting zone (bacteriocin leucocin C) and a 2.2-fold increase in the RFP intensity, compared with wild-type strain [139]. Other industrially relevant strains in the context of heterologous protein expressions, such as *Pseudomonas putida* and *Corynebacterium*, showed equally encouraging outcomes in terms of protein productivity [141] and genetic engineering [142] (Table 2). 

Together, this evidence highlights the advantages of genome-reduced strains in heterologous expression. Generally, overexpression of heterologous genes force-feeds metabolic fluxes and gene expression machineries that are otherwise dedicated to host metabolism to the production of target proteins [28,158]. Subsequent depletion of metabolic precursors and shared translational resources invoke cellular adaptive responses that resemble starvation and amino acid depletion, often manifested in the form of reduced cell biomass, growth retardation, and loss of cell viability [28,158]. Systematic deletion of redundant genes and associated metabolic pathways seems to alleviate the genetic load in the genome-reduced strain, as evident through the increased growth and target protein yield. Second, targeted removal of mutation-causing insertion elements from the genome dramatically increases the genetic fidelity of recombinant expression systems, enabling a long-term, stable recombinant protein expression.

#### 3.2.4. Optimization of Codon Usage 

Another frequent issue in recombinant protein production lies in processing rare codons in the target heterologous genes. Differences in the usage of synonymous codons on the coding regions of the genome have been reported to influence transcriptional and translational features, including translation elongation rates [160], mRNA folding stability [161], protein production rate [161], and protein folding [160]. The effect of codon usage is especially compelling in heterologous protein expression, where incompatibility of codon usage between recombinant genes and expression host (also known as codon bias) has often hampered target protein expression due in large part to a sheer shortage of rare tRNAs or lack thereof [9,162]. It was suggested that codon abundance is positively correlated with the availability of complementary tRNA species, which is then proportional to protein production [9]. The paucity of host tRNA molecules orthogonal to a cluster of codons highly abundant in the target protein could inflict a host of translational errors during heterologous expression, including premature termination (ribosomal stalling) [163], amino acid misincorporation [164], translational frameshifts [165], and decreased protein quality and yield [9,55], which is further aggravated upon overexpression of the target heterologous protein. Efforts to thwart codon-bias-derived expression problems have been computationally optimizing the codon profile of a target gene and selecting for the synonymous codons that best fit the codon usage of the expression host [166]; gene sequence optimization [165]; or co-overexpressing rare tRNA genes accompanied with supplementation of cognate amino acids in the culture media [9]. The latter option has been exploited in commercially available *E. coli* Rosetta(DE3) strain, which contains a plasmid to coexpress tRNA species common in eukaryotic proteins but rare in *E. coli* (Figure 1D and Table 1) [167]. Expression of rare tRNAs accompanied with substitution of *E. coli* Rosetta(DE3) could enhance the overall productivity of industrial enzymes and recombinant protein fragments [55,167], except in some instances, productivity of individual proteins was poorer compared with that of the control strain, suggesting the presence of unknown metabolic burden [55].

The recent demonstration of genome-recoding from bottom-up to yield a fully functional *E. coli* [117] opened a way for a new synthetic biology design for the production of heterologous proteins. The synthetic *E. coli* genome now harbors 59 out of 61 sense codons and resembles otherwise isogenic *E. coli* MDS42 (Table 2) [124]. A total of 18,214 codons composed of two serine synonymous codons UCG and UCA, and the amber stop codon UAG underwent substitution with their synonymous counterparts, leaving three unassigned (blank) codons in the genome. Extended application of the availability of blank codons entailed assignment and incorporation of noncanonical amino acids (NCAAs) in desired genes with the help of aminoacyl-tRNA synthetase complementary to the NCAAs (Figure 2) [29,117], opening the possibility of engineering microbes for the production of a wide, heterogeneous spectrum of proteins [29]. Furthermore, with 57-codon genome underway to elucidate many more aspects of translation [168], it is speculated that designing genome-recoded chassis oriented toward expression of heterologous proteins could be synergized by working in tandem with contemporary troubleshooting designs, tRNA overexpression, and gene sequence optimization to alleviate imminent problems of heterologous protein expression originating from the codon bias. 

#### 3.2.5. Changes in Translation Efficiency 

The abundance of mRNA level ensuing from the overexpression of heterologous gene alone may not necessarily correspond to high protein levels, indicating the presence of post-transcriptional regulations [169,170]. This regulatory mechanism is an extension to codon bias in that the presence of rare codons causes retardation in translation elongation rate [163], and it was shown to be negatively correlated to the abundance of mRNA transcripts and their translation [169]. An investigation into this phenomenon gave rise to the term translation efficiency (TE), which is poised to explain the variation between the abundance of mRNA transcripts and cognate protein molecules [169]. TE provides a measurable means to quantitatively assess how effectively translation is taking place within the mRNA transcripts as presented in the ratio between ribosome-protected mRNA fragment levels (RPF) and the corresponding transcript counts per gene [169]. In the same study, it was demonstrated that mRNA transcripts with variable abundance showed strikingly lesser variations in ribosome occupancy. In other words, translational regulations work to conserve cellular protein abundance by controlling the number of mRNAs actively translated by ribosomes, as featured through ribosome-occupied mRNA transcript counts. Such a mechanism effectively buffers for the variations in mRNA levels, possibly functioning as part of a compensatory mechanism for aberrant mRNA expression and to stably maintain steady-state protein levels. This phenomenon, often referred to as translational buffering, hinders the proper expression of heterologous proteins in prokaryotes [9,163]. However, the molecular basis underlying translational buffering is still under active investigation [169,170,171], rendering it highly challenging to establish strategies for translational-level regulation control. It is only out of hindsight that we know that the interplay of multiple intrinsic [163] and extrinsic [172] factors can, each with differing magnitudes [148,173], pose an influence on translational buffering. These include the sequence on 5′ untranslated region (5′ UTR), action of regulatory proteins, and ribosomal stalling (rare codons). Among the proposed measures to alleviate translational buffering, increasing cellular tRNA availability is regarded as the most feasible solution to mitigate ribosomal pausing [173]. Until recently, a study reported diminished translational buffering in a minimal genome *E. coli* that underwent evolutionary engineering (Figure 2) [148], where the genome-reduced *E. coli* MS56 [127] subjected to adaptive evolution for 800 generations (resulting in eMS57) constantly exhibited higher TE values for mRNAs that share a similar expression level with that of MG1655 control (Table 2). While evolutionary engineering of prokaryotes has been shown to rewire metabolic pathways and reorganize transcriptomes [22], eMS57 showed a rare instance of global-scale remodeling of translatome mediated by adaptive evolution. The authors concluded that the diminished translational buffering in eMS57 was indeed communicated by the translatome-wide remodeling in the process of adaptation, and in principle, this change was driven by an increased availability of ribosome as per the reduced gene counts and a more spacious spatial proximity between neighboring genes that may buffer the expression of one another [174]. Although actual protein productivity of this strain has not been empirically tested, it can be said that the integration of evolutionary engineering with rational genome reduction provides a potentially promising strategy to optimize translational regulation. 

## 4. Conclusion and Perspectives 

Microbial expression systems have been delivering wide varieties of high-quality recombinant proteins in a scalable and cost-effective manner. While incompatibility between microbial translation machineries and eukaryotic protein architecture presents a serious setback to heterologous protein expression, new discoveries and engineering strategies have been put forward to circumvent the problems. Today, catalogs of highly specialized microbial chassis are available for the functional expression of recombinant proteins with specific structural and biochemical demands (Table 1). Despite the efforts, the complex interplay of cellular physiology and recombinant expression pathways still renders the optimization of protein expression a highly challenging task. Recent advances in the systems-level understanding of cellular expression machineries and the wealth of transcriptomic and proteomic data together present an alternative scheme that could possibly mitigate the bottlenecks in recombinant protein expression. 

The shared perspectives of systems and synthetic biology fostered an emerging idea that cells can be made simpler and more efficient by eliminating components of the genome deemed unnecessary for survival. Indeed, precisely designed genome reductions have consistently led to enhanced cellular features including genomic stability, growth rate, biomass, protein productivity, and genetic pliability (more amenable to genetic engineering) in the target microbial cells. For the most part, an increasing number of existing microbial chassis have been genome-streamlined to resolve some of the bottlenecks in heterologous protein expression and to further optimize their performance. Two opposing approaches exist for the construction of reduced genome strains: top-down genome reduction and bottom-up genome synthesis. Despite the shared goal, the two opposing approaches essentially differ with respect to their area of expertise. While the top-down genome reduction offers a pragmatic approach to further improve the functionality of existing cell factories, the current status of the bottom-up synthesis is more inclined toward understanding the fundamental aspects of the minimal genome. Although it is said that the bottom-up genome synthesis may provide new ground to synthesize non-natural proteins for medicinal use, the underlying cost and time to build one such cell do not outweigh the benefits of chemical-based synthesis methods, at least for the near future. Nevertheless, genome-reduction approaches overall represent an ingenious attempt to understand the gene functions and correct for the glitches in genetic makeups that potentially undermine the performance of the cell factories. However, it is to be noted that the genome reduction alone is far from sufficient to tackle every aspect of difficult-to-express protein expression and that the scope of genome reduction in heterologous protein expression is highly specific to certain aspects (i.e., cellular resource reallocation, alleviation of protein toxicity, translation modulation). A carefully designed genome-reduction strategy would open an additional window to further improve the cellular capacity for heterologous protein expression.

## Figures and Tables

**Table 1:** Examples of prokaryote-based heterologous expression systems.

Strain	Defining Features	Reference
*E. coli* (*pgl* cluster)	Expression of *N*-glycosylation pathway of *C. jejuni* origin, catalyzing glycosylation of recombinant proteins where appropriate.	[45]
*E. coli* (human JNK1)	Coexpression of human Jun N-terminal kinase 1 (JNK1) effectively catalyzes recombinant protein phosphorylation	[46]
*E. coli* (*rimJ*)	Use of the native acetylation machinery in *E. coli* to acetylate human proteins.	[47]
*E. coli* (yeast NatA NatB)	Coexpression of yeast-derived NatA NatB acetylation enzymes for amino-terminal acetylation	[48]
*E. coli* (Nε-acetyllysine)	Site-directed incorporation of Nε-acetyllysine using a three plasmid system expressing recoded target genes, suppressor tRNA, and evolutionarily engineered aminoacyl-tRNA synthetase.	[49]
*E. coli* (Chaperone overexpression)	Coordinated co-overexpression of *E. coli* native molecular chaperones—GroEL/ES, DnaK/J/GrpE, IbaA/B, and ClpB—significantly improves the solubility of the recombinant proteins.	[50]
*E. coli* Origami	Facilitates formation of disulfide bonds within the cytoplasmic compartment, through inactivation of thioredoxin and glutathione reductase pathways (Δ*trxB*, Δ*gor*, *aphC*)	[51]
*E. coli* CyDisCo	Introduction of eukaryotic thiol oxidase and disulfide isomerase encourages formation of disulfide bonds within the *E. coli* cytoplasm.	[52]
*E. coli* C41(DE3), C43(DE3)	BL21(DE3) derivative with mutations that confer increased tolerance to toxic membrane proteins.	[53]
*E. coli* Lemo21(DE3)	Harbors a gene expression system that allows fine-tuning of overexpression intensity. Suitable for membrane protein production.	[54]
*E. coli* Rosetta	Alleviates codon-bias by overexpression of tRNA species orthogonal to rare codons in *E. coli*—AUA, AGG, AGA, CGG, CUA, CCC, and GGA.	[55]
*B. subtilis* WB600	Strain that lacks six out of seven extracellular proteases to circumvent host-mediated proteolysis	[56]
*B. subtilis* (*dlt^-^*)	Inactivation of D-anlanylation in *dlt^-^ B. subtilis* increases the availability of folding factors Mg^2+^, Ca^2+^ and Fe^3+^ around the cell membrane microenvironment, alleviating protease-mediated recombinant protein degradation.	[57]
*L. lactis* NZ9000	Microbial expression system that effectively supports the production of various prokaryotic and eukaryotic membrane proteins.	[58]
*Haloferax volcanii*	Halophilic archeon that stably overexpresses seven transmembrane helix proteins such as bacteroiopsins. The transmembrane protein expression machinery can be exploited to express eukaryotic proteins with similar protein topology.	[59,60]
*Geobacillus kaustophilus*	Thermophilic bacteria with an array of heat-stable, sugar-inducible promoters demonstrated soluble expression of heterologous enzymes otherwise insoluble in mesophilic host. With maximal protein yield of 59 mg/L	[61]

**Table 2:** Examples of synthetic genome-reduced strains with defining characteristics beneficial for heterologous protein expression.

Strain	Designation	Genome Reduction	Notable Characteristics	Reference
*E. coli* MG1655	MDS42	663 kbp (14.3%)	IS-free strain with increased stability of exogenous genetic construct. Increased yield of chimeric fusion protein. Improved efficiency of electroporation comparable to commercial DH10B strain. Additional engineering yielded improved recombinant protein productivity	[124]
*E. coli* MG1655	MS56	1068 kbp (23.0%)	IS-free strain with increased stability of exogenous genetic construct. Higher electroporation efficiency.	[127]
*E. coli* MG1655	eMS57	1089 kbp (23.5%)	Evolutionary engineering of MS56 restored the growth on minimal medium. Diminished translational buffering predicted to increase production of recombinant proteins.	[148]
*E. coli* W3110	MGF-01	1030 kbp (22.2%)	Increased growth density.	[125]
*E. coli* W3110	DGF-298	1670 kbp (35.9%)	Higher genome stability, increased growth rate.	[149]
*B. subtilis* 168	MGIM	991 kbp (23.5%)	Small reduction in growth rate, comparable enzyme production.	[135]
*B. subtilis* 168	MBG874	874 kbp (20.7%)	Protein productivity increased up to 2.5-fold. Enhanced nutrient utilization.	[113]
*B. subtilis* 168	PG10	1460 kbp (~36%)	Improved secretory protein production, including that of some of the difficult-to-produce proteins.	[137]
*L. lactis* NZ9000	9k-4	72 kbp (2.8%)	2.2- to 2.5-fold increase in recombinant protein activities. Higher final cell density and growth rates.	[139]
*P. putida* KT2440	EM383	266 kbp (4.3%)	Higher growth rate and final cell density. Showed as much as 41% increase in recombinant protein yield depending on the carbon source used.	[141]
*C. glutamicum* ATCC 13032	MB001	205 kbp (6.2%)	Increased recombinant protein activity and transformation efficiency.	[142]
